# Klotho enhances bone regenerative function of hPDLSCs via modulating immunoregulatory function and cell autophagy

**DOI:** 10.1186/s13018-023-03849-8

**Published:** 2023-06-02

**Authors:** Qingru Niu, Huan Chen, Qianmin Ou, Shuqing Yang, Yingying Peng, Yunyi Xie, Le Yu, Zhilan Cheng, Yang Cao, Yan Wang

**Affiliations:** Hospital of Stomatology, Guangdong Provincial Key Laboratory of Stomatology, Guanghua School of Stomatology, Sun Yat-Sen University, Guangzhou, 510055 China

**Keywords:** Klotho, Human periodontal ligament stem cells (hPDLSCs), Macrophage; Stem cell transplantation, Bone regeneration, Autophagy

## Abstract

**Background:**

Human periodontal ligament stem cells (hPDLSCs) have a superior ability to promote the formation of new bones and achieve tissue regeneration. However, mesenchymal stem cells (MSCs) are placed in harsh environments after transplantation, and the hostile microenvironment reduces their stemness and hinders their therapeutic effects. Klotho is an antiaging protein that participates in the regulation of stress resistance. In our previous study, we demonstrated the protective ability of Klotho in hPDLSCs.

**Methods:**

A cranial bone defect model of rats was constructed, and the hPDLSCs with or without Klotho pretreatment were transplanted into the defects. Histochemical staining and micro-computed tomography were used to detect cell survival, osteogenesis, and immunoregulatory effects of hPDLSCs after transplantation. The in vitro capacity of hPDLSCs was measured by a macrophage polarization test and the inflammatory level of macrophages. Furthermore, we explored autophagy activity in hPDLSCs, which may be affected by Klotho to regulate cell homeostasis.

**Results:**

Pretreatment with the recombinant human Klotho protein improved cell survival after hPDLSC transplantation and enhanced their ability to promote bone regeneration. Furthermore, Klotho pretreatment can promote stem cell immunomodulatory effects in macrophages and modulate cell autophagy activity, in vivo and in vitro.

**Conclusion:**

These findings suggest that the Klotho protein protects hPDLSCs from stress after transplantation to maintain stem cell function via enhancing the immunomodulatory ability of hPDLSCs and inhibiting cell autophagy.

**Supplementary Information:**

The online version contains supplementary material available at 10.1186/s13018-023-03849-8.

## Introduction

Mesenchymal stem cells (MSCs) are multipotent progenitor cells that have the capability of self-renewal, multi-lineage differentiation, and immunoregulation, making them an ideal cell source of tissue engineering and regeneration [1, 2]. Orofacial and periodontal bone tissue losses are major problems to be solved clinically. Human periodontal ligament stem cells (hPDLSCs) are an important source of dental MSCs from the periodontal ligament, and they can be easily obtained using less invasive procedures [3]. Studies have shown that hPDLSCs have an outstanding ability to promote the formation of new bones and periodontal tissues. Moreover, the immunoregulatory ability of hPDLSCs is superior to those of human bone marrow stem cells, which is the essential function of MSC in tissue regeneration progress. As the main candidate stem cells for periodontal tissue regeneration, further researches on the modulatory effects of hPDLSCs have been carried out in recent years [4, 5].

The paracrine process plays an essential role in the mechanism of MSC transplant therapy [6]. MSCs regulate the local microenvironment through paracrine effect, which is considered to be one of the major mechanisms involved in its ability to improve regeneration outcomes. Immune cells, including macrophages, are an integral part of the regenerative process as they exert certain function after sensing extracellular signals such as hypoxia, ischemia, and MSC paracrine signaling [7, 8]. Therefore, the interaction between stem cells and immune cells in the local microenvironment may play a crucial role in modifying the regenerative process [7–9].

However, the direct transplantation of hPDLSCs into the defected area does not always result in adequate recovery [10]. This is because local transplantation of MSCs is often associated with poor cell survival due to the harsh environment at the site of the damaged tissue [11]. The low oxygen and nutrient supply in damaged site decreases MSC survival and proliferation rates [12]. Under such circumstances, local production of reactive oxygen species increases rapidly and beyond the scavenging capacity of antioxidant defenses, resulting in oxidative stress. Consequently, oxidative stress impairs the stemness and immunoregulatory capacity of MSCs, leading to failed tissue regeneration [13]. However, it is currently unknown whether the secretome from hPDLSCs that have been injured by such processes would affect the local immune cells.

Autophagy-related pathways are associated with the immunosuppressive effect of MSCs [14]. Exosomes of mesenchymal stem cells (MSC-exos) can regulate autophagic markers such as Beclin-1 and microtubule-associated protein light chain 3 (LC3) in rat renal tubular epithelial cells, promoting the anti-inflammatory response [15]. Moreover, MSCs can transport depolarized mitochondria into macrophages. MSC-exos increase the expression of the M2 receptor CD206 and decrease pro-inflammatory effects through oxidative phosphorylation of macrophages in acute respiratory distress syndrome models [16].

Therapeutic strategies that improve the antioxidative ability and maintain the vitality and immune-regulatory capacity of MSCs are imperative. To improve the therapeutic effect, conventional strategies such as genetic modifications, hormones, and growth factors have been employed with some degree of success [17, 18]. Our previous study demonstrated the protective effects of the recombinant Klotho protein on hPDLSCs under oxidative conditions in vitro, by restoring the mitochondrial antioxidant system [19]. Klotho expressed in Osx+-mesenchymal progenitors promotes osteogenesis during bone repair, and inhibits inflammatory bone resorption [20]. Inflammatory condition or oxidative stress was reported to activate autophagy. Insufficient or excessive autophagy can lead to the occurrence and development of aging-related diseases, such as vascular disease, muscular dystrophy and diabetes [21, 22]. Recent studies have suggested that Klotho regulates autophagy in various tissues [21]. However, whether the Klotho protein can provide anti-oxidative protection to hPDLSCs in vivo is unknown, and the role of Klotho in affecting the hPDLSC immune-regulatory and autophagic-regulatory systems remains unclear.

Therefore, this study further investigated whether Klotho protein is capable of protecting the vitality and therapeutic effects of hPDLSCs after transplantation, including the survival rate of post-transplanted stem cells and the fate and functions of hPDLSCs based on bone regenerative outcomes in vivo. Furthermore, we examined macrophage polarization and the regulatory mechanisms that hPDLSCs exerted in vitro and in vivo to determine whether Klotho preserves the immunomodulatory effects of hPDLSCs after transplantation.

## Materials and methods

### Cell culture and treatment

hPDLSCs were isolated and cultured as previously described [19]. They were obtained from 12 healthy premolars with orthodontic demands (12–20 years old, 6 males and 6 females). This study was approved by the Ethics Committee of the Affiliated Stomatological Hospital of Sun Yat-sen University (ERC-[2016]-46). For this study, hPDSLCs were acquired from passages 3–5. The hPDLSCs were seeded in 6-well plates at a density of 1 × 10^5^ cells/well. Recombinant Klotho protein (PeproTech, Rocky Hill, NJ, USA) was added to the culture medium and incubated for 24 h. According to a previous study, 100 ng/mL Klotho was used in this study for the optimum protective effect. The hPDLSCs were pretreated with 100 ng/mL Klotho for 24 h.

### Transplantation of cells in a rat model of cranial bone defect

The animal experiments were conducted in accordance with the approved principles and procedures of the Ethics Committee of Zhongshan School of Medicine on Laboratory Animal Care (SYSU-IACUC-2022-001065). Male Sprague Dawley (SD) rats, weighing 180–220 g and aged 8 weeks, were procured from the Guangdong Medical Laboratory Animal Center (Guangzhou, China). The rats were divided into groups of six for the experiment. Anesthesia was induced with 1% pentobarbital administered through intraperitoneal injection. The skin on the rats' heads was prepared and incised, and the periosteum was carefully dissected to expose the cranial bone. Two critical-size cranial defects with a diameter of 6 mm were created on each side of the cranium to evaluate new bone formation. The hPDLSCs were pretreated with 100 ng/mL Klotho for 24 h and then cultured with fresh medium before collected for implantation. hPDLSCs pretreated with or without Klotho were transplanted into the defects using Matrigel scaffolds (Corning, NYC, USA), simultaneously. 2 × 10^6^ cell/50 µL cell suspension was mixed with 100 µL Matrigel on ice for each defect and can be used after changing into solid phase in room temperature. The cranial defects treated with only Matrigel scaffolds but no cells were defined as Blank control. Finally, absorbable sutures were used to suture the incisions (*n* = 6).

### Evaluation of cell viability after transplantation

To evaluate the viability of hPDLSCs in vitro, the expression of c-Caspace-3 and total Caspase-3 were detected using immunofluorescence staining at 0 and 24 h cultured with or without Klotho treatment. To evaluate the viability of hPDLSCs after transplantation in vivo, tissue samples from cranial bone defects were collected 3 days after the operation. The soft tissue samples were fixed in a 10% formalin solution and embedded in paraffin. Paraffin sections were dewaxed at 58 °C for 20 min and hydrated with gradient concentration of ethanol. The sections were soaked in citrate repair solution for antigenic thermal repair. 0.1% TritonX-100 (prepared in PBS) was added to cover the tissues for 30 min, and then the sections were closed in 5% BSA solution for 2 h at 25 °C. Rabbit anti-human Ki-67 (1:400; Cell Signaling Technology, Waltham, MA, USA)/mouse anti-human HNA antibody (HNA) (1:200; Millipore, Bedford, MA, USA) and rabbit anti-human c-Caspase-3 antibody (1:400; Cell Signaling Technology, Waltham, MA, USA)/mouse anti-human HNA antibody were added separately, and incubated overnight at 4 °C. The samples were then conjugated to secondary antibodies containing fluorescent of both species for 1 h at 25 °C. Finally, the nuclei were stained with Hoechest (Life Technologies, USA) and observed in laser confocal microscope (Carl Zeiss, Oberkochen, Germany).

### Autophagy activity evaluation of transplanted cell

Tissues in cranial bone defects were collected 3 d after the operation to assess PDLSC autophagy activity after transplantation. The samples were treated via the method mentioned in the “Evaluation of cell viability after transplantation” section, after being blocked with 5% (w/v) bovine serum albumin/PBS for 2 h at 25 °C, and incubated with LC3 antibody (1:200; Abcam, Cambridge, MA, UK) and HNA (1:200; Millipore, Bedford, MA, USA) overnight at 4 °C, and further conjugated to Alexa Fluor555 or Alexa Fluor488 (1:500; EMAR, Beijing, China) as secondary antibodies for 1 h at 25 °C. Finally, the nuclei were counterstained using DAPI (1:100; Abcam, Cambridge, MA, UK). Fluorescent images were observed and recorded using a laser confocal microscope (Carl Zeiss, Oberkochen, Germany).

### Micro-computed tomography (CT) examination

The tissues of the cranial defect area were collected from each group 12 week post-transplantation and Micro-CT (SCANCO μCT50, Muttenz, Switzerland) scans were performed to observe new bone formation in rat cranial defects. Mimics software (version 17.0, Materialise, Leuven, Belgium) was used to analyze the selected region of bone defect and export the analytical outcome including the measurement of bone volume and bone density of the newly formed bone.

### Histological evaluation

The specimens were evaluated histologically after undergoing μCT scanning. To perform histological analysis, the specimens were fixed in a 10% formalin solution, decalcified with a 10% EDTA solution (pH 7.4), and subsequently embedded in paraffin. 5-μm-thick sections were stained with hematoxylin and eosin (H&E) as well as Masson's trichrome stains to facilitate histological evaluation. To assess the local macrophage polarization, iNOS (1:400; Cell Signaling Technology, Waltham, MA, USA) and Arginase1 (Arg1) (1:400; ProteinTech Group, Chicago, IL, USA) antibodies were applied at the bone defect site. Additionally, immunohistochemical staining was performed using antibodies against BSP, IL-6, IL-1β and IL-10. The digital slice scanner (Leica, Wetzlar, Germany) was used to capture images. Tartrate-resistant acid phosphatase (TRAP) staining was performed on cryosections using the Acid Phosphatase, Leukocyte (TRAP) Kit (Millipore Sigma, USA) according to the manufacturer's instructions. Percent of the positive area was measured by ImageJ (Germany).

### Macrophage culture and conditioned medium treatment

To explore the effect of Klotho on the immunoregulatory capacity of hPDLSCs, macrophages were cultured in different hPDLSC-conditioned medium (PDLSC-CM). At first, the hPDLSCs were seeded onto 10 cm dishes at a density of 5 × 10^5^ cells/well and treated with or without 100 ng/mL Klotho protein for 24 h. Then the supernatants were collected and mixed with fresh DMEM (Gibco, GrandIsland, USA) containing 10% FBS in a 1:1 ratio, named as PDLSC-CM-control and PDLSC-CM-Klotho. To test the effect of Klotho and CMs on the viability of Raw264.7 macrophages, cells were treated with different concentrations of Klotho or CMs for 24 h, and then CCK8 assay was performed. The murine RAW264.7 macrophage line was cultured in DMEM, supplemented with 10% heat-inactivated FBS. After the adherent culture using DMEM for 16 h, the RAW264.7 macrophages were cultured in different PDLSC-CM for 24 h, and then with M1 inducers (1 µg/mL LPS and 20 ng/ml IFN-γ) for another 24 h at 37 °C. Meanwhile, the macrophages that were only cultured in DMEM throughout the course were used as negative controls (uninduced group), and macrophages that were treated without PDLSC-CM but only with M1 inducers for the last 24 h were made as positive controls (induced group).

### Flow cytometric assessment of surface markers

The RAW264.7 macrophages (3 × 10^5^) were cultured in 24-well plates in DMEM overnight (16 h), and then cultured via the method mentioned in the “Macrophage culture and conditioned medium treatment” section. The macrophages were then washed twice with PBS, harvested using a scraper, collected through centrifugation, and maintained on ice. Subsequently, the cells were resuspended in 100 µL PBS, in tubes, blocked with CD16/32 Fc block (1:200; BioLegend, CA, USA) for 15 min on ice. The cells were then washed with PBS, collected, and maintained on ice. Then, a mixture of 0.2 µL anti-mouse BV421-CD163 and 0.2 µL anti-mouse APC/cyanine7 IA/IE antibodies (BioLegend, CA, USA) in 100 µL PBS was added to each tube, and rested for 30 min, in darkness, on ice. The cells were then washed and resuspended in 250 µL PBS and recorded using a flow cytometer (BD LSRFortessa flow cytometry, State of New Jersey, USA). The data were analyzed using the FlowJo 10.8.1 software.

### Quantitative real-time reverse transcription polymerase chain reaction (qRT-PCR)

Total RNA samples were isolated from the hPDLSCs and RAW264.7 macrophages, using an Ultrapure RNA kit (CWBIO, Beijing, China). Reverse transcription was performed using a Reverse Transcriptase M-MLV Kit (Takara, Shiga, Japan), according to the manufacturer’s instructions. PCR was performed using the SYBR Green Kit (GenStar, Beijing, China) and the LightCycler® 480 Real-Time PCR System (Roche, Basel, Switzerland). The amplification conditions were set as follows: 95 °C for 10 min, 40 cycles of denaturation at 95 °C for 15 s, annealing at 60 °C for 20 s, and final extension at 72 °C for 20 s. The gene expression levels were calculated using the 2^−ΔΔCt^ method (*n* = 3). The primer sequences used are listed in Table 1.

**Table 1:** Primer sequences used in quantitative real-time reverse transcription polymerase chain reaction

Species	Gene target	Sequence
Human	LC3	Forward: 5′–GATGTCCGACTTATTCGAGAGC–3′
		Reverse: 5′–TTGAGCTGTAAGCGCCTTCTA–3′
	ATG5	Forward: 5′–AAAGATGTGCTTCGAGATGTGT–3′
		Reverse: 5′–CACTTTGTCAGTTACCAACGTCA–3′
	Beclin1	Forward: 5′–GGTGTCTCTCGCAGATTCATC–3′
		Reverse: 5′–TCAGTCTTCGGCTGAGGTTCT–3′
	PINK1	Forward: 5′–GGAGCGAGATCCCTCCAAAAT–3′
		Reverse: 5′–GGCTGTTGTCATACTTCTCATGG–3′
	GAPDH	Forward: 5′–CTCCCTAACCGTCTCCGCT–3′
		Reverse: 5′–GGCTCTCCGCCTGTTTTTC–3′
Mouse	IL-1β	Forward: 5′–TGCCACCTTTTGACAGTGATG–3′
		Reverse: 5′–TGATGTGCTGCTGCGAGATT–3′
	IL-6	Forward: 5′–TGATGGATGCTACCAAACTGG–3′
		Reverse: 5′–TTCATGTACTCCAGGTAGCTATGG–3′
	iNOS	Forward: 5′–AATCTTGGAGCGAGTTGTGG–3′
		Reverse: 5′–CAGGAAGTAGGTGAGGGCTTG–3′
	Arg1	Forward: 5′–CAGAAGAATGGAAGAGTCAG–3′
		Reverse: 5′–CAGATATGCAGGGAGTCACC–3′
	18sRNA	Forward: 5′–GCAATTATTCCCCATGAACG–3′
		Reverse: 5′–GGGACTTAATCAACGCAAGC–3′

### Western blot analysis

The cells were lysed and quantified using the Pierce BCA protein assay kit (Thermo Scientific, Waltham, MA, USA). Then, the protein samples were separated using 10% SDS-PAGE and transferred to PVDF membranes (Millipore, Bedford, MA, USA). The membranes were then blocked by 5% skimmed milk for 1 h and then incubated with primary antibodies at 4 °C, overnight. Finally, the membranes were incubated with horseradish peroxidase (HRP)-conjugated secondary antibodies (Emar, Beijing, China) or fluorescent secondary antibodies (LI-COR, Biosciences, Lincoln, NE, USA) for 1 h at 25 °C. Antibodies against iNOS (1:1000; Cell Signaling Technology, Waltham, MA, USA), Arg1 (1:5000; ProteinTech Group, Chicago, IL, USA), LC3 (1:1000; Abcam, Cambridge, UK), Autophagy-related protein 5 (ATG5) (1:1000; Abcam, Cambridge, UK), Beclin-1 (1:1000; Cell Signaling Technology, Waltham, MA, USA), PINK1 (1:600, ProteinTech Group, Chicago, IL, USA) and GAPDH (1:1000; Zenbio, Chengdu, China) were used in this study. The relative density of the protein bands was analyzed using the ImageJ software and normalized to GAPDH (*n* = 3).

### Statistical analysis

Statistical analysis was performed using the SPSS 20.0 software package (SPSS Inc., Chicago, IL, USA). One-way analysis of variance (ANOVA) was conducted, and the post hoc Bonferroni test was performed for multiple comparisons. Statistical significance was set at *P* < 0.05.

The experiments were performed in triplicate, and the data were expressed as mean ± standard deviation.

## Results

### Klotho protected the viability of the hPDLSCs after transplantation in vivo

**Fig. 1:**
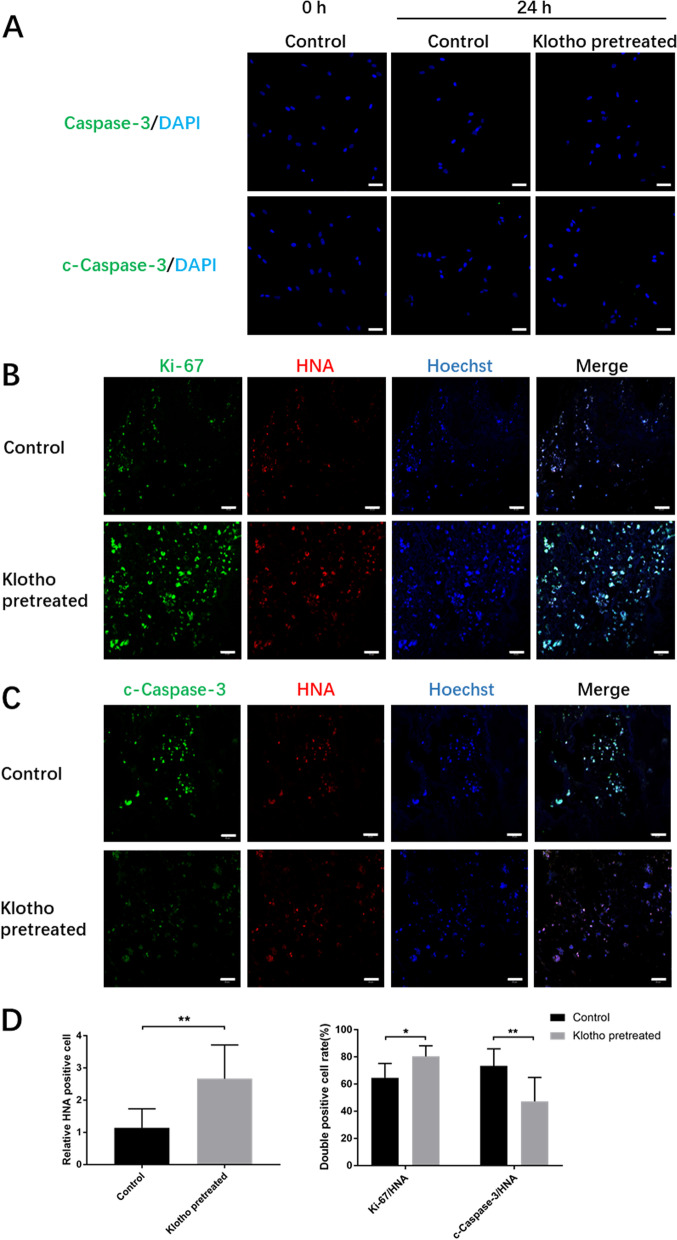
Effect of Klotho pretreatment on hPDLSC survival at the transplantation site. **A** Immunofluorescence images of total Caspace-3 and c-Caspase-3 expression in hPDLSCs. **B** Immunofluorescence images of proliferating hPDLSCs (green and red overlap). **C** Immunofluorescence images of apoptotic hPDLSCs (green and red overlap). **D** HNA-positive, Ki67/HNA and C-caspase-3/HNA double-positive cell rates (%) in each group. Scale bar = 50 µm (**A**) and 20 µm (**B**, **C**). *P < 0.05, **P < 0.01

The isolated cells exhibited colony-forming abilities and expressed mesenchymal stem cell markers while lacking epithelial cell markers, as confirmed by immune-fluorescence and flow cytometry analyses (Additional file 1: Fig. S1A, B, D). The cells were also able to differentiate into osteoblasts and adipocytes (Additional file 1: Fig. S1C), indicating that they were MSCs. The cells in 0 h and 24 h cultured with or without Klotho treatment weakly expressed or even did not express c-Caspace-3 and total Caspase-3 (Fig. 1A). It indicated that the cells we transplanted had good viability. To assess the impact of Klotho protein on survival ability of hPDLSCs post-transplantation, the transplanted cells were labeled with an anti-HNA antibody, while Ki-67 and c-Caspase-3 markers were used to label proliferating and apoptotic cells, respectively. The Klotho-pretreated groups exhibited significantly higher numbers of HNA-positive and Ki-67/HNA double-positive cells than the control group, whereas the number of c-Caspase-3/HNA double-positive cells was significantly lower in the Klotho-pretreated group (Fig. 1B–D). These findings indicate that Klotho treatment can enhance cell proliferation and reduce apoptosis in hPDLSCs three days after transplantation in vivo.

### Klotho pretreatment promotes bone regenerative abilities of hPDLSCs after transplantation in rat cranial bone defects

**Fig. 2:**
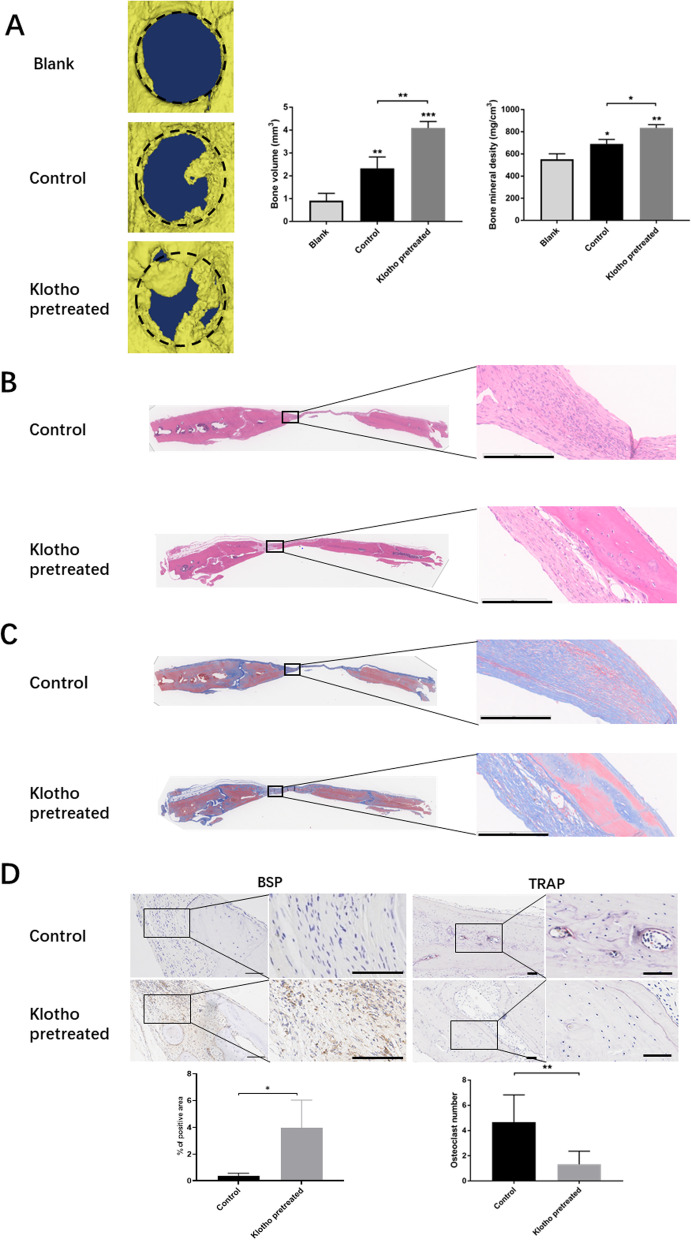
Effect of Klotho pretreatment on hPDLSC bone regenerative ability at the transplantation site. **A** Micro-CT and quantitative analysis of the new bone regeneration areas in vivo. **B** HE staining analysis of the new bone growth in the cranial defects. **C** Masson’s trichrome staining analysis of the new bone growth in the cranial defects. **D** Expression levels of BSP and TRAP staining in each group, and quantitative data (BSP expression and TRAP-positive cells). Scale bar = 200 µm (**B**, **C**) and 100 µm (**D**). *P < 0.05, **P < 0.01

Assessment of bone tissue regeneration by hPDLSCs was performed by analyzing cranial bone tissue samples post-transplantation. Compared to the control and blank groups, the Klotho pretreated group displayed a significantly larger area of new bone calcification within the bone defect, with increased bone volume and mineral density (Fig. 2A). Histological examination using HE staining revealed more extensive bone regeneration in the Klotho pretreated group (Fig. 2B), while Masson's trichrome staining demonstrated intermingling of bone tissue and collagen fibers in the newly formed bone (Fig. 2C). Importantly, new bone regeneration was significantly greater in the Klotho-pretreated group than in the control group, strongly suggesting that Klotho promotes the osteogenic ability of hPDLSCs and accelerates osseous defect healing in vivo. These findings represent a significant step towards developing therapeutic approaches for bone tissue repair using stem cell-based regenerative medicine. Moreover, BSP staining showed a higher osteogenic outcome in new bone tissue of the Klotho group, and TRAP staining revealed increased osteoclastic activity in the control group, compared to the Klotho group (Fig. 2D).

### Klotho pretreatment modulated the immune-regulatory effect of hPDLSCs on macrophage polarization and local anti-inflammatory proteins, in vivo

**Fig. 3:**
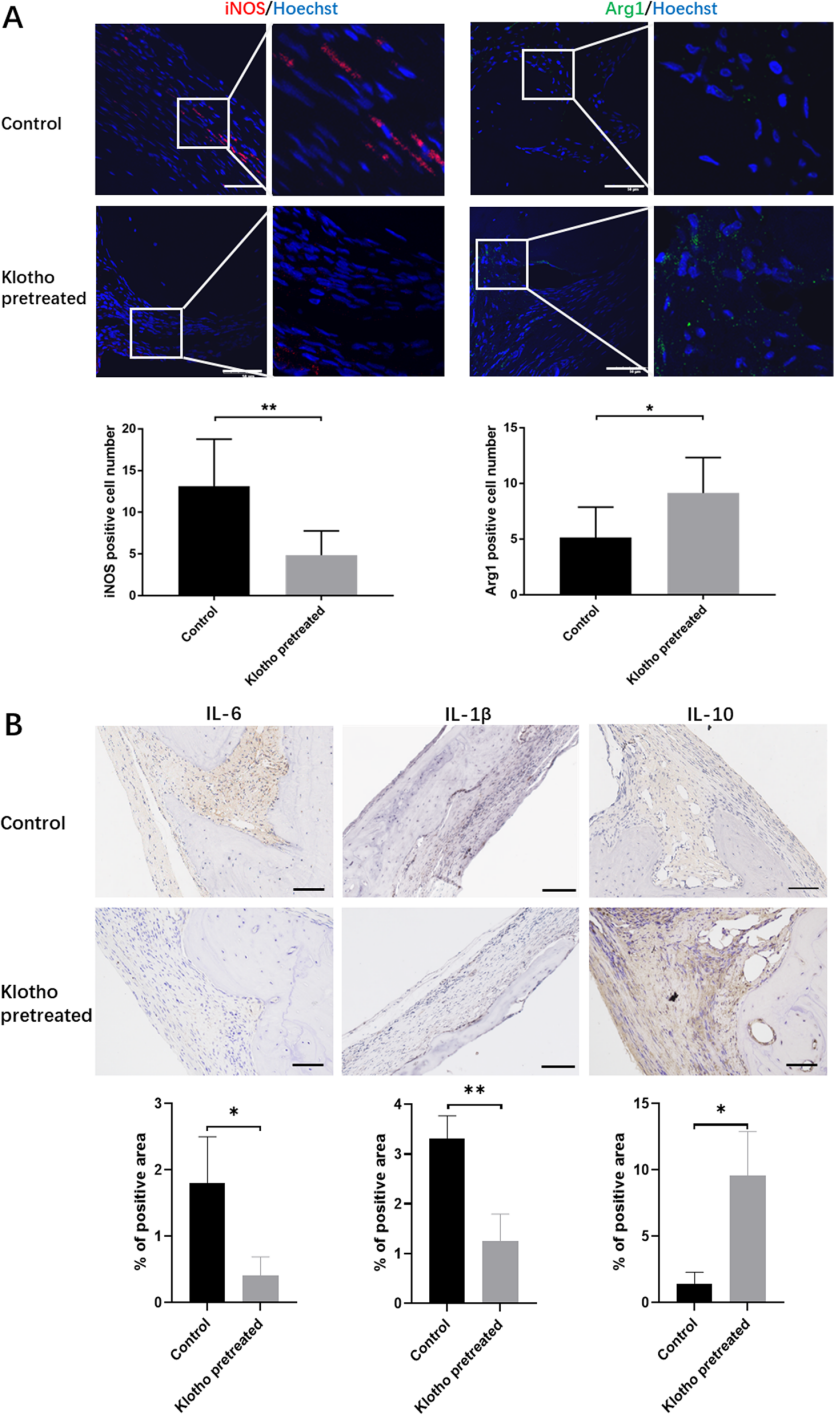
Effect of Klotho pretreatment on hPDLSC immune-regulatory ability at the transplantation site. **A** M1 macrophages (iNOS positive cells) and M2 macrophages (Arg1 positive cells), and the number of iNOS and Arg1 positive cells in the bone tissue of each group. **B** Expression levels of inflammatory factors (IL-6, IL-1β and IL-10) in the bone tissues of the rats in each group, and the percent of positive area of each group; Scale bar = 50 µm (**A**) and 100 µm (**B**). *P < 0.05, **P < 0.01

To assess the influence of transplanted hPDLSCs on macrophage polarization, we assessed the presence of the M1 macrophage marker iNOS and M2 macrophage marker Arg1 at the bone defect site 12 weeks after hPDLSCs transplantation. Macrophage polarization affects the inflammation stage and local microenvironment in bone reconstruction, and M2 macrophages inhibit inflammation and promote tissue regeneration [23, 24]. The macrophages in the Klotho-pretreated group showed the highest expression of the M2 phenotype and the lowest expression of the M1 phenotype, which was conducive to the repair of the defective bone (Fig. 3A).

In addition, we measured the expression of IL-6, IL-1β and IL-10 at the bone defect site to assess the influence of hPDLSCs on the local immuno-microenvironment. The decreased expression of the inflammatory factor interleukin-6 (IL-6), inflammatory factor interleukin-1β (IL-1β) and increased expression of the anti-inflammatory factor interleukin-10 (IL-10) indicated that Klotho pretreated hPDLSCs might exert anti-inflammatory effects in vivo (Fig. 3B), suggesting that Klotho pretreated hPDLSCs might upregulate multifunctional therapeutic cytokine expression with low proinflammatory properties and regulate the immuno-microenvironment via paracrine effects.

### Klotho promotes the regulation of hPDLSCs on macrophages by stimulating M2 polarization and suppressing M1 polarization

**Fig. 4:**
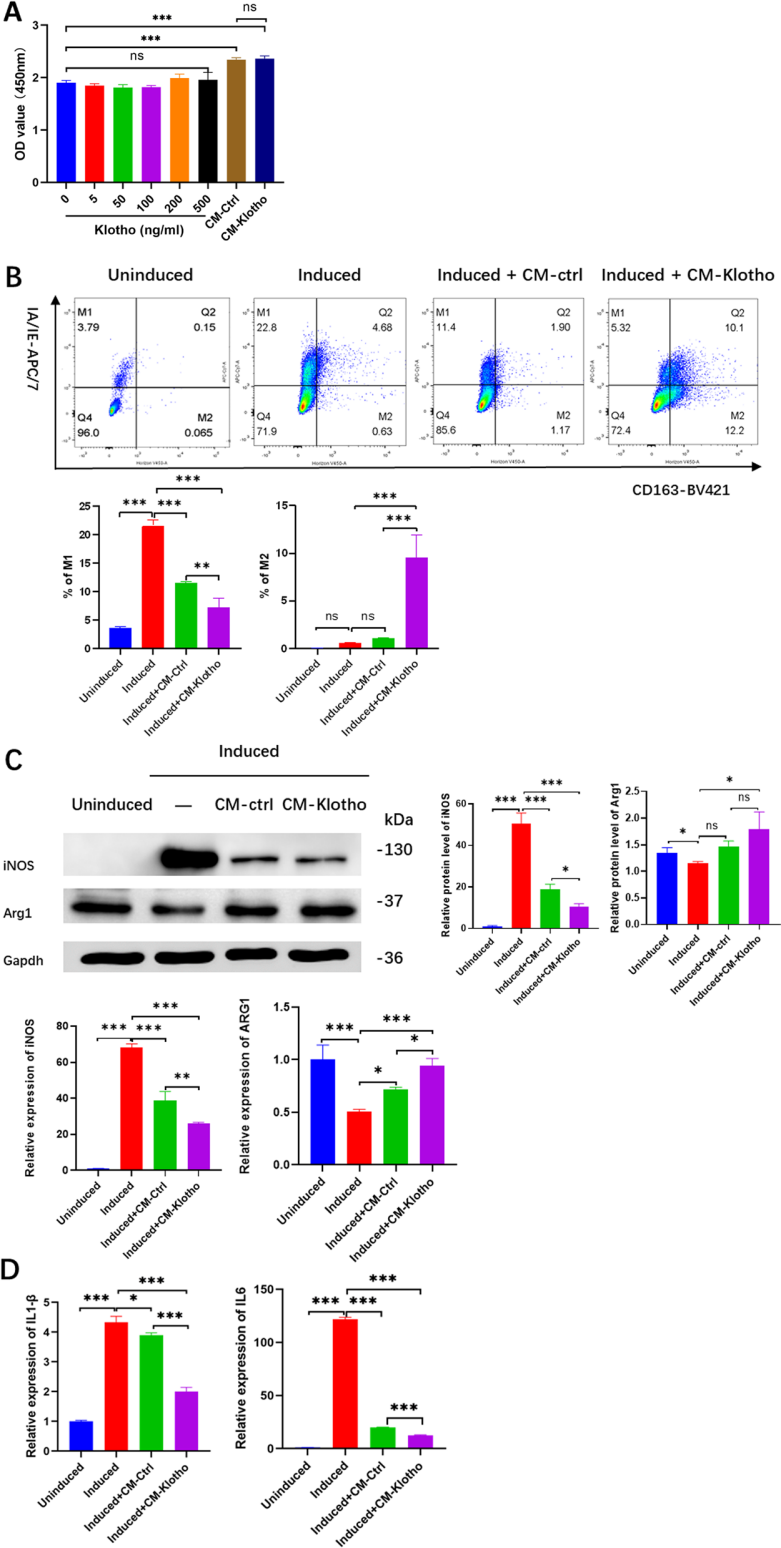
Klotho-pretreated hPDLSC conditioned medium has an anti-inflammatory effect on Raw264.7 macrophages, in vitro. **A** CCK8 assay of macrophage viability. **B** Flow cytometric analysis of macrophage polarization and the percentages of M1 and M2 subtypes. **C** Western blot analysis and RT-qPCR of macrophage polarization. **D** RT-qPCR analysis of IL-1β and IL-6 gene expression levels in macrophages. *P < 0.05, **P < 0.01, ***P < 0.001, ns: not significant. Raw264.7 macrophages were treated according to the groups described in "Flow cytometric assessment of surface markers" section and were divided into 4 groups: Uninduced; Induced (short for M1 induced); Induced + CM-Ctrl (short for hPDLSC-CM-control + M1 induced); Induced + CM-Klotho (short for hPDLSC-CM-Klotho + M1 induced)

Raw264.7 macrophages was treated with different concentrations of Klotho or CMs for 24 h, followed by CCK8 assay to detect the cell viability. The results showed that different concentrations of Klotho had no obvious effect on Raw264.7 cells viability. Both of CMs could promote cell viability, but there was no significant difference between the two types of CMs (Fig. 4A). The macrophages were treated and cultured with an hPDLSC-conditioned medium, in vitro to explore the effect of Klotho on the immunoregulatory capacity of hPDLSCs. The polarization of Raw264.7 to the M1 type was inhibited in the hPDLSC-CM-control group. The hPDLSCs in the hPDLSC-CM-Klotho group played a stronger immunomodulatory role than those in the hPDLSC-CM-control group, which significantly inhibited the polarization of RAW264.7 into the M1 type, and promoted its polarization to the M2 type (Fig. 4B, C). The results showed that Klotho pretreatment of hPDLSCs inhibited the polarization of macrophages to the M1 type and promoted the polarization of macrophages to the M2 type through the paracrine CM. The RT-qPCR results showed that the expression levels of the proinflammatory cytokines IL-1β and IL-6 in Raw264.7 macrophages were significantly increased after the induction of M1. The mRNA expression level of IL-6 in Raw264.7 was reduced in the hPDLSC-CM-control group, but no obvious effect on the mRNA expression level of IL-1β was observed. The expression levels of IL-1β and IL-6 were significantly reduced in the hPDLSC-CM-klotho group (Fig. 4D), which was consistent with the flow cytometry results.

### Klotho inhibits the hPDLSCs cellular hyperactivation of autophagy in vivo and vitro

Normal cell autophagy is crucial for tissue homeostasis. Insufficient or excessive autophagy can lead to the occurrence and development of diseases. Hyperactivation of autophagy may promote cell death, especially when excessive consumption of cytoplasmic components occurs during sustained autophagy. Oxidative stress at the MSC transplantation site has also been reported to activate autophagy [21, 22, 25]. Therefore, moderate autophagic activity is crucial for stem cell homeostasis and regeneration. In our study, autophagy markers were detected to evaluate autophagy activity in hPDLSCs pretreated with the Klotho protein.

**Fig. 5:**
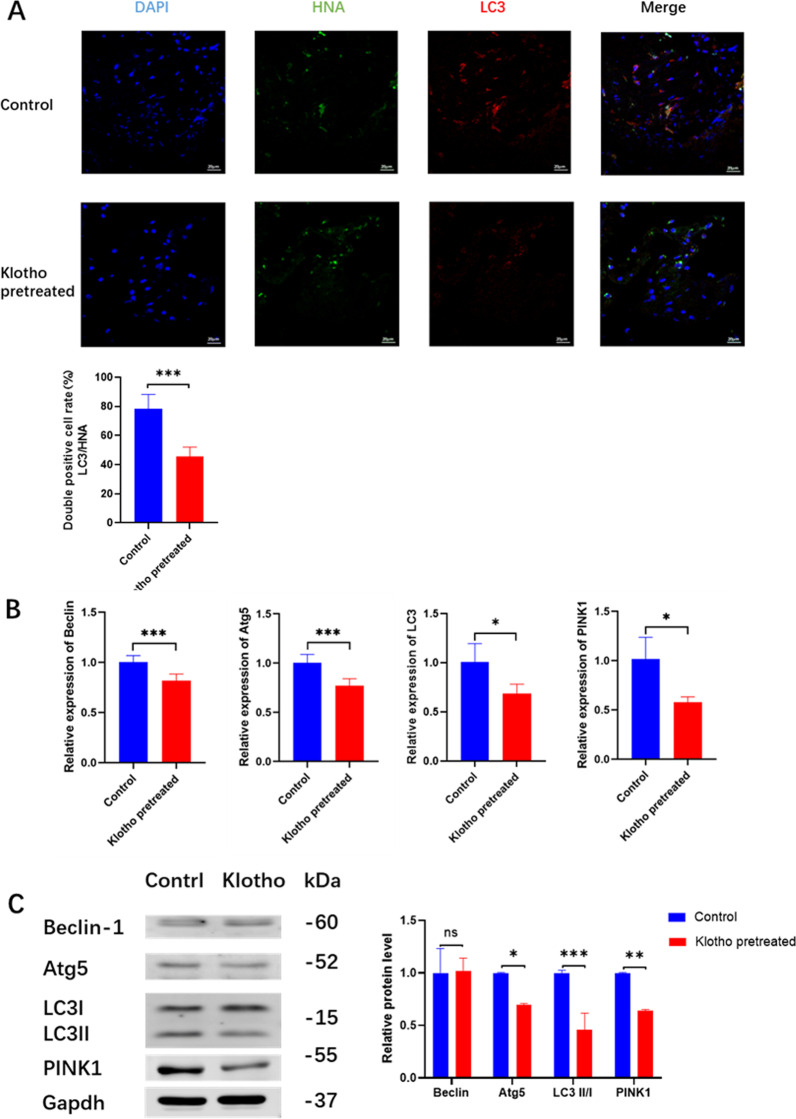
Effect of Klotho pretreatment on hPDLSC cellular autophagy activity in vivo and vitro. Scale bar = 20 µm. **A** Immunofluorescence images of HNA (green fluorescence), LC3 (red fluorescence), and DAPI (blue fluorescence). **B** RT-qPCR analysis of cellular autophagy gene expression levels in macrophages. **C** Western blot analysis and quantification of cellular autophagy activity. *P < 0.05, **P < 0.01, ***P < 0.001, ns: not significant

In vivo, after transplantation, hPDLSCs were quantified as the number of HNA-positive cells, and the intensity of red light represented the LC3 level in hPDLSCs (Fig. 5A). The results showed that the LC3/HNA double-positive cells in the Klotho-pretreated groups were significantly less than those in the control group (Fig. 5A), which shows that autophagy activity in local transplanted cells is abnormally elevated and Klotho pretreatment maintains the autophagy activity of hPDLSCs at a stable lower level. In vitro, western blot and RT-qPCR analysis showed a lower autophagy activity in hPDLSCs after Klotho treatment, which implied that Klotho exerted a stabilized function in hPDLSCs, protecting the cells from the harsh environment after transplantation (Fig. 5B, C).

## Discussion

The therapeutic effects of transplanted oral MSCs, such as hPDLSCs, have been studied and prepared for application in oral diseases for decades [26]. Evidence from preclinical studies suggests that MSCs exert tissue-regenerative effects mostly through the secretion of various paracrine factors and immunomodulation [27, 28]. However, the tissue environment at the transplantation site may critically influence the effects and properties of MSCs. In the present study, we investigated whether pretreatment with Klotho protein could protect against MSC apoptosis and sustain anti-inflammatory activity to improve the regenerative effects of hPDLSCs.

MSCs function under the premise that the transplanted MSCs survive for a certain period. Early-stage survival of MSCs is crucial for the success of MSC therapy. The results showed that the number of remaining hPDLSCs in the Klotho-pretreated group was higher than that in the control group, and the number of remaining hPDLSCs in the proliferative state was also higher. These results suggest that Klotho pretreatment of hPDLSCs in vitro can enhance the cell activity and reduce early-stage cell apoptosis after transplantation in vivo. Massive cell apoptosis in stem cell transplantation is an important factor that directly negatively affects tissue regeneration after transplantation, and the survival rate is closely related to the effect of tissue regeneration [29].

At 12 weeks after transplantation, results showed that Significant new bone formation was observed at the edge of the bone defect in the Klotho-pretreated group. This may be related to the improvement in hPDLSCs survival after transplantation by Klotho pretreatment. According to recent studies, transplanted stem cells can directly differentiate into osteoblasts and osteocytes to participate in bone formation and can also act on host cells by secreting cytokines and extracellular vesicles to enhance the tissue regeneration ability of host cells and form new tissues [30]. Moreover, understanding the paracrine effects of MSCs involved in the tissue regeneration process is important for developing efficient and safe regenerative medicine applications [31]. Immunohistochemical staining showed that the expression of BSP in Klotho-pretreated tissues was higher than that in the control group, and the expression of IL-10 was significantly increased. The early markers of osteogenesis include BSP and RUNX2, they are important factors of extracellular matrix [32, 33]. Our result indicates that Klotho-pretreated hPDLSCs may promote onset osteogenesis by regulating bone homeostasis and inhibiting osteoclast activity in tissues. Changes in IL-10 levels are related to the surface molecules of activated macrophages. IL-10 can reduce antigen presentation and inhibit the expression of proinflammatory factors such as IL-6 by downregulating the expression of the major histocompatibility antigen II on the surface of immune cells [34].

Macrophages play a key role in tissue repair and inflammation. In response to microenvironmental changes, they can be polarized into M1 phenotype or an ‘M2-like’ state. Nitric oxide produced by M1 macrophages is significant to inhibit microbicidal activity and cell proliferation, while arginase1 produced by M2 macrophages is benefit for tissue remodeling and formation [35]. In our study, Klotho pretreatment of hPDLSCs polarized more macrophages into the M2 phenotype and inhibited the polarization of macrophages to the M1 type. M2 macrophages play a different role in different phages of tissue regeneration. The macrophages promote the reconstitution of vascular networks during the early, then facilitate collagen synthesis in the wound bed, and eventually form granulation tissue, providing a temporary condition for cells to migrate, communicate, and proliferate [36, 37]. This explains the mechanism by which Klotho maintains osteogenic ability. Klotho regulates calcium and phosphorus metabolism. In vivo, Klotho's effect can prevent abnormal calcification and inhibit poor osteogenic or cartilage inflammation [38] without directly promoting bone differentiation.

Macrophages often exhibit a proinflammatory M1 phenotype in the early stages of repair but change to an anti-inflammatory M2 phenotype in the later stages of wound healing. There is also a continuous phenotype between them, and the subtype transition of macrophages plays a key role in the transition between the inflammatory and proliferative phases [39]. MSCs can mediate the polarization of macrophages in a variety of ways, and macrophages can respond to microenvironmental factors in tissues [40]. Conditioned medium after MSCs culture, which contains growth factors, cytokines, microRNA (miRNA), and other small molecular weight signal cues, have great regenerative and immunomodulatory potential [41]. Pretreating MSCs during in vitro culture is able to modulate the composition of the MSCs' secretome [42]. A recent study found that force could stimulated hPDLSC autophagy, and CM from the stimulated cell would increase M1 macrophage polarization via the AKT signaling pathway [43]. The results of this study confirmed that hPDLSCs exhibit immunomodulatory functions in vitro, and inhibit the polarization of M1 macrophages. However, when immune cells are stimulated to be in an inflammatory state, their immunomodulatory function is limited, and the degree of inhibition of macrophage inflammation is also limited. After pretreatment with Klotho, the immune regulatory function of hPDLSCs was effectively promoted, and hPDLSCs exerted a strong immune regulatory ability.

Autophagy is the main cellular mechanism for degrading and recycling intracellular proteins and organelles under different physiological and pathological conditions, although excessive autophagy leads to tissue loss and aging [44, 45]. In MSC, Autophagy may affect their ability to differentiate and to affect the activation, proliferation, and functions of immune cells [46]. Emerging evidence has confirmed that inhibition of autophagy partially rescued MSC-mediated therapeutic efficacy in the restraint stress condition [47]. Another study showed that MSC-exos can upregulate the expression of autophagic markers such as ATG5 and ATG7 [15]. However, the hyperactivation of autophagy may promote cell death [25]. In our study, we investigated LC3 expression in Klotho-pretreated hPDLSCs after transplantation and found a lower LC3 expression, indicating a temporary protective effect through the suppression of autophagy in transplanted cells. Some studies unveil that the inhibition of autophagy of MSCs can increase their immunosuppressive effects on T cell-mediated experimental autoimmune encephalomyelitis [48]. Our results showed that Klotho inhibited autophagy of hPDLSCs and enhanced their immunoregulatory effects on macrophages at the same time. In previous study, Klotho promotes bone regeneration via rejuvenating aged stem cells and modulating autophagic flux [49]. In addition, the mutation of Klotho gene in mice can shortens the lifespan and causes skeletal muscle atrophy [49]. We observed suppressed ATG5 and LC3 expression in Klotho-pretreated hPDLSCs. The suppression of autophagic markers observed in this study implies that Klotho may reduce the overexpression of autophagy proteins in hPDLSCs which led to the loss of cell homeostasis, thus protecting cells from stress, after transplantation. MSCs are an efficient intervention to diminish cell death, and downregulating the over-induction of the autophagy pathway [50]. The Klotho protein seems to protect and promote the functions and immunoregulatory effects of MSCs through an autophagy-modulation way. Moreover, the attainable curtailing effects on MSCs can result in fostering biogenesis pathways, such as mitochondrial renewal [51], which may explain the PINK1 changes in hPDLSCs after Klotho treatment. Further investigations, such as mitophagy-related progress, and the relation between immunoregulatory effects and autophagy-inhibition of hPDLSCs should be conducted to verify these hypotheses. Overall, the strategy of Klotho administration on stem cell protection in bone tissue engineering can be a promising method to improve the cell anti-stress ability and the cell function in regenerative therapy.

## Conclusions

Thus far, the findings of our study imply that pretreatment with recombinant human Klotho protein could improve cell survival after hPDLSC transplantation and enhance the ability of hPDLSCs to promote bone regeneration. Furthermore, Klotho pretreatment promoted stem cell immune-regulatory effects on macrophages and modulated autophagy activity in vivo and in vitro. These findings suggest that the Klotho protein enhances the immunomodulatory ability of hPDLSCs and protects stem cells from stress after transplantation to maintain stem cell function via inhibiting cell autophagy. These findings suggest that the Klotho protein protects hPDLSCs from stress after transplantation to maintain stem cell function via enhancing the immunomodulatory ability of hPDLSCs and inhibiting cell autophagy.

## Supplementary Information

**Additional file 1: Figure S1** Characterization of hPDLSCs. (A) Representative images of a single colony-forming unit of hPDLSCs at 12 d. (B) Immunofluorescence staining demonstrated that hPDLSCs expressed vimentin but not cytokeratin. (C) The ability of hPDLSCs to differentiate into multiple cell types, as demonstrated by Alizarin red and Oil red O staining under specific differentiation conditions for osteoblasts or adipocytes. (D) Cell surface markers of hPDLSCs were detected by flow cytometry. Scale bars: 100 µm (A), 50 µm (B), 200 µm (C1), 20 µm (C2).

## Data Availability

The data supporting the findings of this study are available from the corresponding author upon reasonable request.

## Abbreviations

hPDLSCs: Human periodontal ligament stem cells
MSCs: Mesenchymal stem cells
MSC-exos: Exosomes of mesenchymal stem cells
LC3: Microtubule-associated protein light chain 3
SD: Sprague Dawley
HNA: Anti-human HNA antibody
H&E: Hematoxylin and eosin
Arg1: Arginase1
TRAP: Tartrate-resistant acid phosphatase
hPDLSC-CM: Human PDLSC-conditioned medium
HRP: Horseradish peroxidase
ATG5: Autophagy-related protein 5
ANOVA: One-way analysis of variance
IL-6: Inflammatory factor interleukin-6
IL-10: Anti-inflammatory factor interleukin-10
