# Supplementary figures and images for: Klotho enhances bone regenerative function of hPDLSCs via modulating immunoregulatory function and cell autophagy
Source: J Orthop Surg Res. 2023 Jun 2;18:400. doi: 10.1186/s13018-023-03849-8 (PMC10236596; doi:10.1186/s13018-023-03849-8)

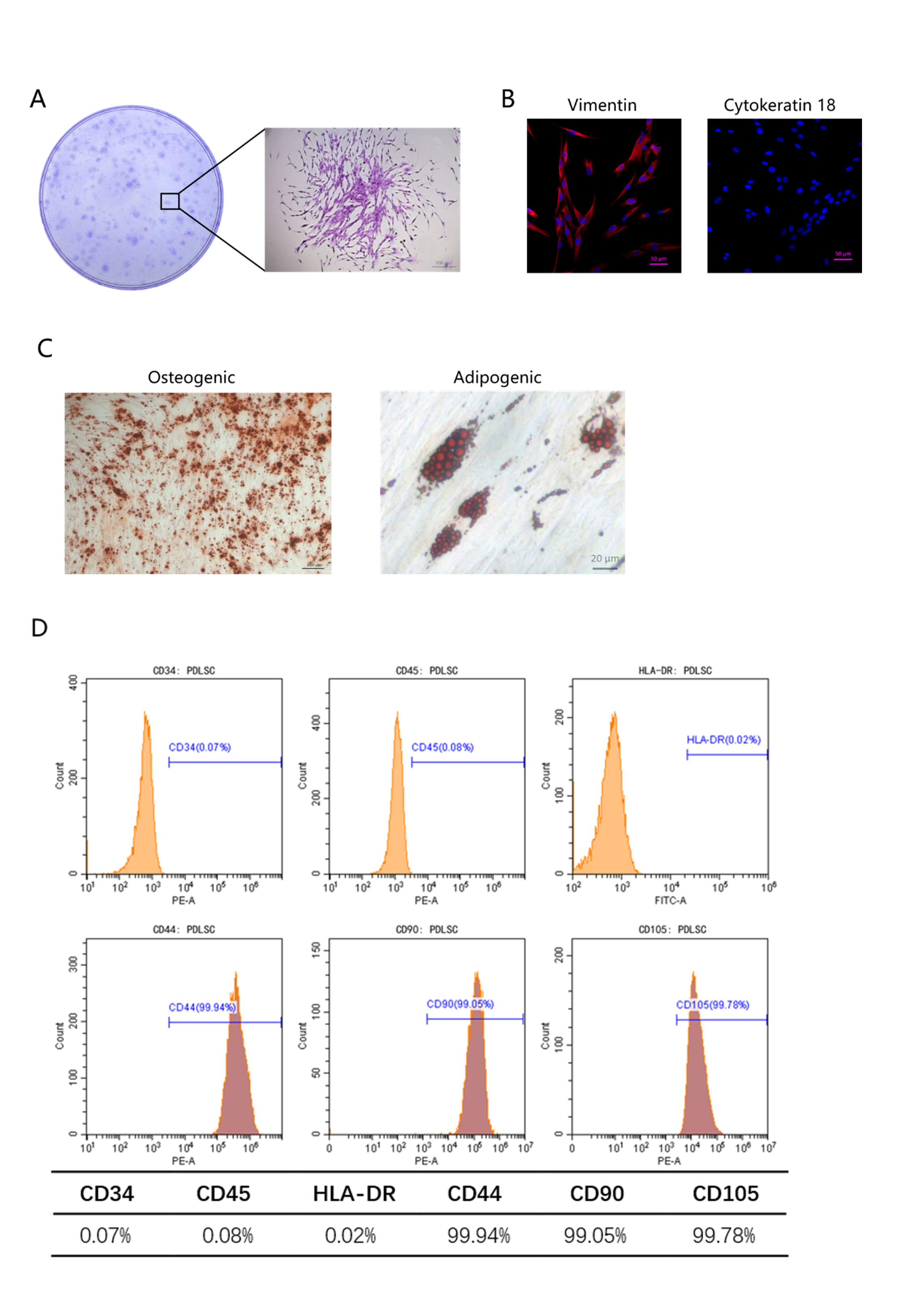

Supplement: Supplementary file 1 — Additional file 1: Figure S1 Characterization of hPDLSCs. (A) Representative images of a single colony-forming unit of hPDLSCs at 12 d. (B) Immunofluorescence staining demonstrated that hPDLSCs expressed vimentin but not cytokeratin. (C) The ability of hPDLSCs to differentiate into multiple cell types, as demonstrated by Alizarin red and Oil red O staining under specific differentiation conditions for osteoblasts or adipocytes. (D) Cell surface markers of hPDLSCs were detected by flow cytometry. Scale bars: 100 µm (A), 50 µm (B), 200 µm (C1), 20 µm (C2). [file 13018_2023_3849_MOESM1_ESM.tif]
